# The Word Frequency Effect on Saccade Targeting during Chinese Reading: Evidence from a Survival Analysis of Saccade Length

**DOI:** 10.3389/fpsyg.2017.00116

**Published:** 2017-02-06

**Authors:** Yanping Liu, Ren Huang, Yugang Li, Dingguo Gao

**Affiliations:** ^1^Department of Psychology, Guangdong Provincial Key Laboratory of Social Cognitive Neuroscience and Mental Health, and Guangdong Provincial Key Laboratory of Brain Function and Disease, Sun Yat-sen UniversityGuangzhou, China; ^2^Key Laboratory of Behavioral Science, Institute of Psychology, Chinese Academy of SciencesBeijing, China

**Keywords:** Chinese reading, word frequency effect, eye-movement control, survival analysis, computational modeling

## Abstract

Our study employs distributional analysis (i.e., survival analysis) to examine how the frequency of target words influences saccade lengths into and out of these target words in Chinese reading. The results of survival analysis indicate the survival curves in the high- and low-frequency conditions diverge for a short saccade length, with more than 80% of the lengths of incoming and outgoing saccades being larger than the divergence points. These results as well as simulations using the novel Dynamic-adjustment Model of saccadic targeting (Liu et al., 2016) are consistent with previous mean-based results and provide more precise information to support this novel model. The implications for saccade target selection during the reading of Chinese are discussed.

## Introduction

What information influences saccades during reading? It is well-known that low-level cues (e.g., interword spaces) can guide eyes to somewhere near the center of words in spaced languages such as English (Rayner, 1979, 1998). Little is known, however, about whether and how high-level information (e.g., word frequency) modulates saccade landing positions or saccade lengths in reading. In unspaced languages, such as Chinese, which do not have explicit cues to demark word boundaries, it is particularly unclear what information can help readers select saccade targets. In this paper, we will adopt a novel method—the distribution analysis—to examine how high-level information such as word frequency impacts on saccade targeting during the reading of Chinese.

Although previous experiments tend to support that low-level information such as word length (or interword spaces) has the primary responsibility for where to fixate next (O'Regan, 1979, 1980; Rayner, 1979; Pollatsek and Rayner, 1982; Morris et al., 1990; Vitu, 1991; Rayner et al., 2001), some studies concentrating on the processing difficulties in fovea and parafovea provide new insights. For example, previous experiments found that the frequency of parafoveal words can influence the length of saccades entering these words in some unspaced languages, such as Chinese (Liu et al., 2015) and Uighur (Yan et al., 2014). Similarly, the same parafoveal word frequency effect also appears in some spaced languages, such as English (Rayner et al., 2006) and Finnish (Hyönä and Pollatsek, 1998, 2000). Moreover, the frequency of foveal words can also influence saccade length when exiting them. The saccades out of high-frequency words are longer than those out of low-frequency words in both unspaced languages (Wei et al., 2013; Li et al., 2014; Liu et al., 2016) and spaced languages (Rayner et al., 2004; White and Liversedge, 2006). The foveal word frequency effect is due to the foveal processing difficulty, which can influence parafoveal processing and then modulate the saccade length for these parafoveal words (see Liu et al., 2015).

In contrast with the aforementioned results, the dominant view is that high-level information influences saccades in another way. A considerable number of experiments present that word frequency only influences a few long saccades that try to skip the next words (Schotter et al., 2014) rather than the majority of saccades that try to land near the center of the next word (Dunn-Rankin, 1978; Rayner, 1979; Inhoff and Rayner, 1986; McConkie et al., 1988; Vitu et al., 1990; Rayner et al., 1996; Reingold et al., 2012). Generally, the skipping rate increases 4% with parafoveal words in the high-frequency condition compared to the low-frequency condition in spaced languages (e.g., Henderson and Ferreira, 1993; Inhoff and Topolski, 1994; Rayner and Fischer, 1996; Rayner and Raney, 1996; Rayner et al., 1996; see Table 1 for detailed comparisons in Brysbaert and Vitu, 1998) as well as unspaced languages (e.g., Yan et al., 2006; Liversedge et al., 2014). Similarly, some experiments indicate that other high-level information, such as word predictability, influences saccades in the same manner. Word predictability only impacts on word skipping for a few saccades (Ehrlich and Rayner, 1981; Rayner et al., 2004, 2011) rather than landing position on the next word for the majority of saccades (Rayner et al., 2001, 2004; but Lavigne et al., 2000). The skipping rate overall increases ~9% for parafoveal words in the high-predictability condition compared to the low-predictability condition in spaced languages (e.g., Ehrlich and Rayner, 1981; Vonk, 1984; Balota et al., 1985; Schustack et al., 1987; Inhoff and Topolski, 1994; Brysbaert and Vitu, 1995; Rayner and Well, 1996; see Table 2 for detailed comparisons in Brysbaert and Vitu, 1998), and at most increases 13% in unspaced languages (e.g., Rayner et al., 2005). These various perspectives regarding how high-level information affects saccade targeting may be due to previous researchers addressing the launch site in different ways. In a new study of Chinese reading, after controlling launch sites carefully, Liu et al. (2016) displayed a robust result that word frequency had an effect on saccade length and on landing position of saccades entering and exiting a word.

Although increasing evidence supports the results that the frequency of parafoveal words influences saccade lengths and landing positions upon entering them, the conclusion is still obscure because the reported frequency effects are relatively small. For example, the length of saccades exiting high-frequency words are 0.5 letters longer than those exiting low-frequency words when reading spaced languages (Rayner et al., 2004). The saccades exiting high-frequency words are just 0.1–0.2 characters longer than those exiting low-frequency words in Chinese reading (Wei et al., 2013). One possible explanation of the small effect is that the frequency effect may appear to slightly elongate long saccades to skip the next words. The few increased skipping saccades only can lead to a small effect (referred as *Slight-skipping Account*). Another possible explanation is that the frequency gradually influences the majority forward saccades in a continuous manner instead of through discretized skipping (referred as *Gradual-adjusting Account*). In this circumstance, although the word frequency affects the majority of forward saccade lengths, it is still difficult to observe a large effect because the adjustment is subtle. The traditional mean-based method appears to be a central tendency but ignores the detailed difference from the data distribution. Therefore, it is less likely to differentiate both above accounts through the mean-based analysis.

In contrast of the small mean-based frequency effect on saccade length, both above accounts can predict different saccade length distributions. As supposed, the *Slight-skipping Account* stipulated that word frequency only appears in a few long saccades which try to skip next word, but the *Gradual-adjusting Account* stipulated that word frequency can appear in short saccades. Thereby, distributional analysis might be more suitable to explore word frequency effect on saccade lengths in current research. Fortunately, recent empirical efforts have employed distributional analyses such as survival analysis to examine eye-movement data (i.e., fixation duration; Staub, 2011; White et al., 2011a,b; Reingold et al., 2012; Sheridan and Reingold, 2012; White and Staub, 2012). This method provides a novel way to examine eye movement data and can reveal more precise information.

Survival analysis is one of the most robust methods to examine the distribution of data by calculating its survival rate. In the medical field, *survival rate* refers to the percentage of people in a treatment who are alive at a given time after diagnosis (e.g., percentage of cancer patients alive each year after being diagnosed with the disease). In the context of fixation duration, the percentage of survival at a given time *t* is the percentage of fixations with a duration >*t*. Reingold et al. (2012) used this method to examine the time course of word frequency effects on the first-fixation duration during reading. They calculated separate survival curves for high- and low-frequency words first and then examined when both survival curves began to diverge (called the divergence point). Consequently, the divergence point provides an estimate of the earliest effect of the word frequency variable and the proportion of fixations longer than this divergence point. Compared with fixation duration, in the context of saccade length, survival rates can be calculated as the percentage of saccades that have lengths greater than *x* using small incremental values of *x*. By calculating survival rates in this way, it is possible to observe where the survival curves begin to diverge in high- and low-frequency conditions and how many saccades are longer than the divergence point, further providing precise information to reveal how word frequency influences saccade targeting.

Therefore, the present study investigates how word frequency influences the distribution of saccade lengths and then distinguishes the aforementioned *Slight-skipping Account* and *Gradual-adjusting Account* based on how word frequency influences saccade length in Chinese reading. We will use the aforementioned survival analysis to examine survival curves for the distributions of saccade lengths that go into and out of the target words and then calculate the divergence point for the high- and low-frequency curves. If word frequency affects saccade length through contributing a higher skipping rate as the *Slight-skipping Account* stipulated, the word frequency effect only can be observed in a few long saccades that attempted to skip next words (the general skipping for two-character words and the word frequency effect on them are only ~10 and 4%, respectively; see Yan et al., 2006). In this situation, the survival curves between the high- and low-frequency conditions will diverge in a long saccade length (i.e., >2 characters, which is the minimal length to skip a 2-character word), and the proportion of saccades longer than the divergence point will be small (i.e., <<50% or proximity to the skipping rate as mentioned above). If word frequency affects the majority of forward saccades gradually as the *Gradual-adjusting Account* stipulated, however, the divergence point will appear in a short saccade length (i.e., <2 characters), and the proportion of saccades longer than the divergence point will be relatively large (i.e., >>50%). In current research, the data were derived from an eye-tracking experiment and a computational simulation using the Dynamic-adjustment Model of saccadic targeting in Chinese reading (Liu et al., 2016). The experiment by Liu et al. explored how foveal and parafoveal processing influence saccade lengths by manipulating target word frequency and preview validity. In their analysis, they adopted a mean-based method to examine the data and found the target word frequency influenced the length of saccades into and out of the target words; however, the frequency effect in their experiment was small. In this paper, we will reanalyze their data through distribution analysis to observe how word frequency impacts on saccade targeting during the reading of Chinese. To avoid any disruption that may be introduced by the display changes, we only adopt the data in valid preview condition.

## Methods

### Participants

Thirty-six undergraduate students (23 males) recruited from universities in Beijing were paid 30 yuan to participant in the experiment. All participants were native speakers of Chinese. All of them had normal or corrected-to-normal vision, and all were naïve to the purpose of the experiment.

### Materials and design

**Figure 1:**
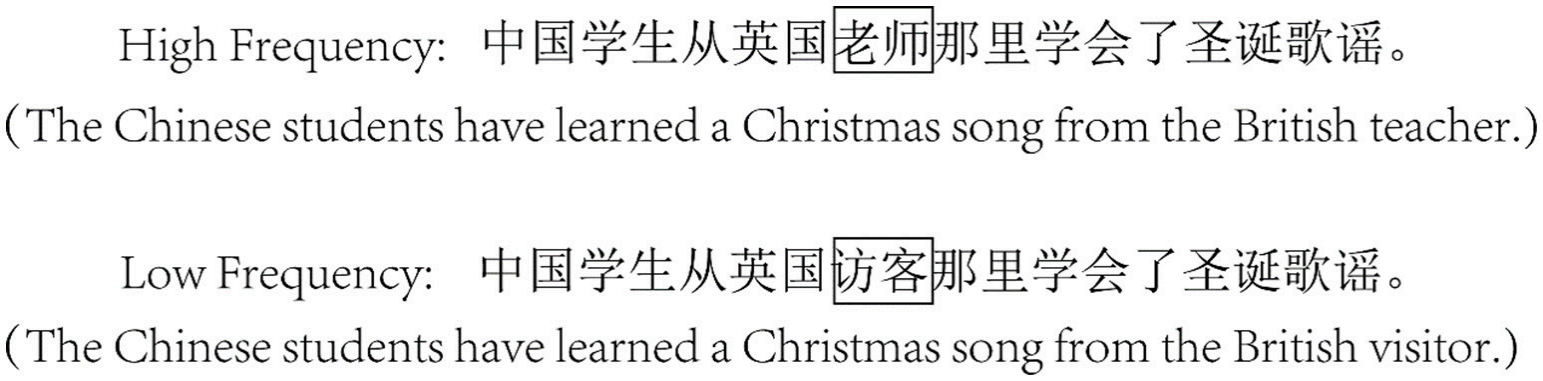
**Examples of the stimuli used in the experiment (target words are indicated by solid lines for illustrative purposes)**.

In Liu et al. (2016), the experiment consisted of a 2 (target-word frequency: high vs. low) × 2 (target-word preview validity: valid vs. invalid) within-subjects design. The materials contained 160 sentence frames. Each frame embedded a high-frequency word (*M* = 121.5 per million; *SD* = 98.5) or a low-frequency word (*M* = 2.17 per million; *SD* = 1.53) in the same location properly (see Figure 1). All target words were two characters long. Ten native Chinese speakers who did not participate in the experiment rated the predictability of the target words. They read the text prior to the target word and wrote a word that would appear following the text. The predictability of each target word was <0.1. Another 20 native Chinese speakers rated the naturalness of the sentences on a 5-point scale (1 means “completely not natural,” 5 means “completely natural”). The naturalness score of each sentence was higher than 3, and the naturalness in high-frequency and low-frequency conditions were no different (*p* > 0.05). As indicated, although the original experiment by Liu et al. used the gaze-contingent boundary paradigm (Rayner, 1975) to manipulate the preview validity of the target words, our paper focuses on the natural reading condition (i.e., the sentences displayed naturally so that readers could extract target word information prior to fixating this target word). For each participant, 80 remaining trials for high- and low-frequency conditions were used in this paper. The participants read equal numbers of sentences in each condition according to the counter-balance design.

### Apparatus

Stimuli were displayed on a 21-in. CRT monitor (SONY Multiscan G520) with a resolution of 1024 × 768 pixels and a 150-Hz refresh rate. All characters were rendered in Song 20 font. The presentation was controlled by an OpenGL-based Psychophysics Toolbox 3 (Brainard, 1997; Kleiner et al., 2007), which incorporates the EyeLink Toolbox extensions (Cornelissen et al., 2002) in Matlab (2013a). Using this configuration, display changes can be controlled precisely and require ~10 ms to complete. Eye movements were recorded using a SR-Research Eyelink 1000 eye tracker (upgraded to 2000 Hz; Kanata, ON, Canada) sampling at a 1000-Hz rate. Participants were seated 58 cm from the monitor. At this distance, one character subtended ~1° of the visual angle. A chin rest was used to minimize head movements. Viewing was binocular, but only the right eye was recorded.

### Procedure

Participants were instructed to answer comprehension questions in the experiment. Calibration and validation were conducted before the experiment. The participants conducted 15 practice trials, which were not included in our analysis. Next, they completed the experiment trials. In each trial, a drift check appeared in the middle of the screen first. If the drift check passed, a fixation box (1° × 1°, the size of a character) appeared at the first character of the sentence. The sentence was displayed on the screen after they successfully fixated the box. The participants read the sentence silently and used the button box to terminate the sentence display. One-third of the trials then displayed a comprehension question. The participants pressed the button (Microsoft SideWinder Game Pad) to answer the question and then began the next trial. If the drift check indicated more than 0.4 degrees of error or the fixation box did not trigger, then recalibration and revalidation were conducted. Furthermore, the participant was recalibrated at regular intervals.

## Empirical results

**Figure 2:**
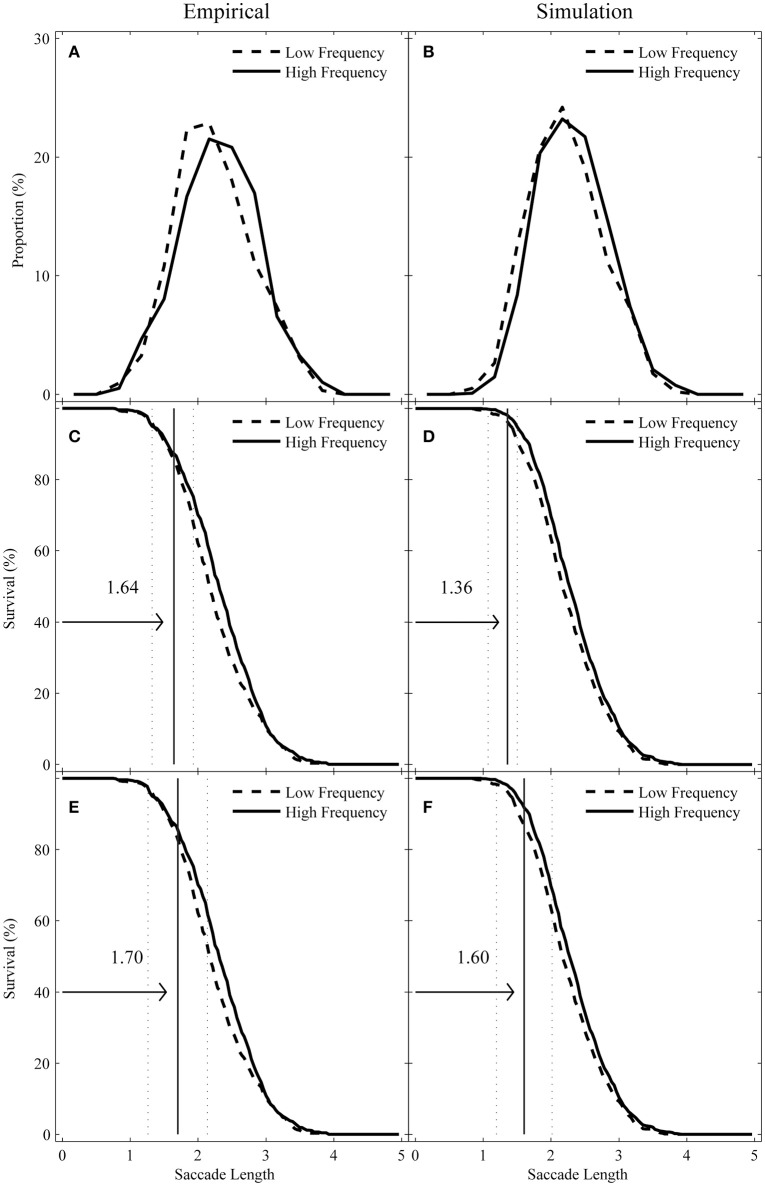
Observed **(A)** vs. simulated **(B)** incoming-saccade length distributions as a function of target-word frequency, along with their corresponding survival curves by using the Confidence Interval DPA procedure **(C,D)** and the Individual Participant DPA procedure **(E,F)**. The vertical solid lines mark the divergence point estimate and the dotted lines represent the 95% confidence interval or the standard deviation of individual participants in their respective panels.

**Figure 3:**
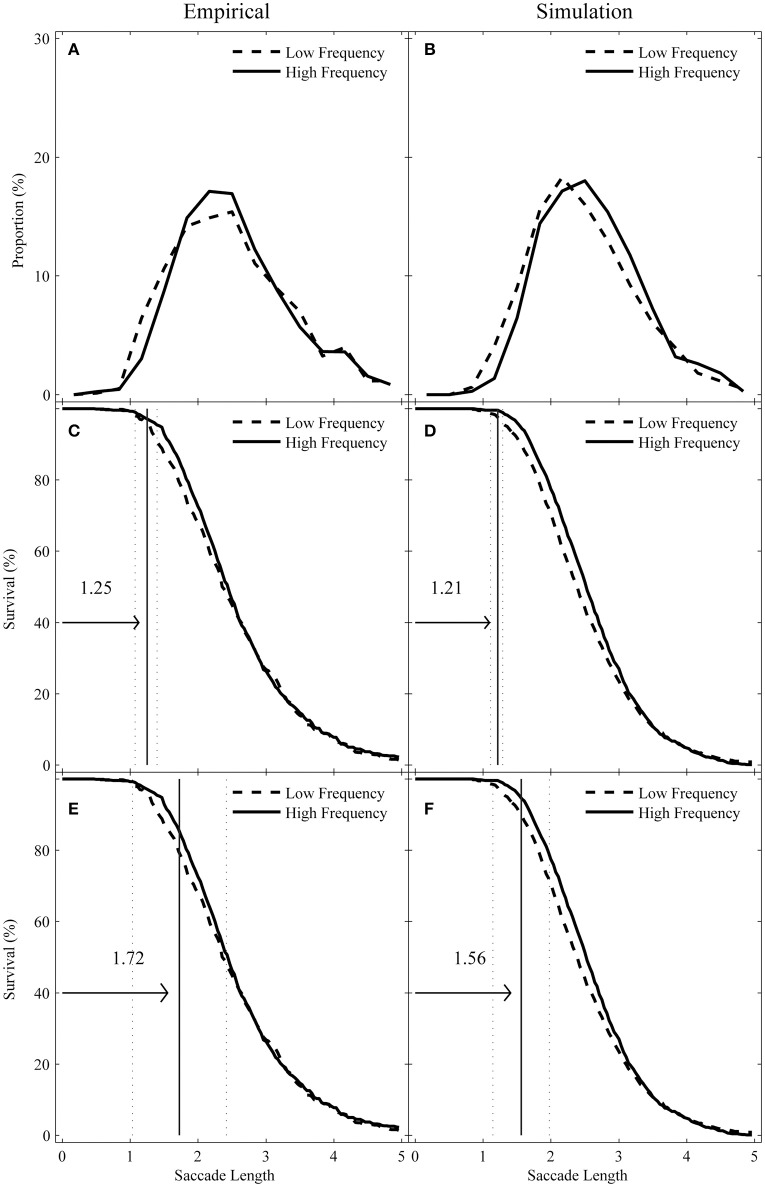
Observed **(A)** vs. simulated **(B)** outgoing-saccade length distributions as a function of target-word frequency along with their corresponding survival curves using the Confidence Interval DPA procedure **(C,D)** and the Individual Participant DPA procedure **(E,F)**. The vertical solid lines mark the divergence point estimate and the dotted lines represent the 95% confidence interval or the standard deviation of individual participants in their respective panels.

In this study, we explored how word frequency influences the saccade lengths entering and leaving target words through distribution analysis. We reported the following two corresponding eye-movement measures during first-pass reading: (1) *incoming-saccade length*, or the length of any saccade landing on the target word from a prior word, and (2) *outgoing-saccade length*, or the length of first progressive saccade that was launched from the target word and that resulted in a fixation to the right of the target word. First, we described the frequency distribution for incoming- and outgoing-saccade length distribution. To do this, we calculated the proportion of incoming- and outgoing-saccade lengths within each successive saccade-length bin (one-third of a character) over the range of 0–5 characters for each participant. These values were averaged across participants to generate the distributions, as shown in Figures 2A, 3A.

Then, we used survival analysis to explore the divergence point for the high- and low-frequency conditions. To do this, we first calculated the percentage of saccades with lengths larger than *x* (*x* was equal to 1 pixel or 1/28 character) for each bin of width *x*. This was performed for each participant and condition and then averaged across participants. The survival curves are shown in Figures 2C,E for incoming-saccade lengths and in Figures 3C,E for outgoing-saccade lengths. Then, we used a bootstrap re-sampling procedure (Efron and Tibshirani, 1994) to conduct the divergence point analysis (DPA). To warrant the reliability of these divergence points (e.g., whether these estimates are different across high- and low-frequency conditions), we used the Confidence Interval DPA to compute confidence intervals for divergence point estimates and the Individual Participant DPA for individual participants. Both procedures were modified from the original definition (Reingold et al., 2012), which was recommended to improve the reliability (and the two modified procedures performed better than the original procedure under conditions of low statistical power) by Reingold and Sheridan (2014). The Confidence Interval DPA and the Individual Participant DPA procedures can be considered supplementary as they contribute unique information about the reliability of these divergence point estimates (see Reingold and Sheridan, 2014 for detailed introduction).

To run the Confidence Interval DPA procedure, 1000 iterations of random resampling of saccades for each participant and condition were used. Thus, the divergence point estimate was defined as the first 1-pixel bin in a run of five consecutive bins in which the survival rate in the high-frequency condition was at least 1.5% greater than the survival rate in the low-frequency condition for each iteration. Then, the 95% confidence interval was defined by the 25th and 975th values in the ranked divergence point estimates from the smallest to the largest values across the 1000 iterations. The median of the 1000 sorted divergence point values was defined as the divergence point estimate for the sample.

To run the Individual Participant DPA procedure, 1000 iterations of random resampling of saccades for each participant were performed separately. For each of the 1000 bootstrap iterations, 1200 saccades for a given participant were randomly sampled with replacement from the respective pool of saccades corresponding to the high- and low-frequency condition. Both sets of 1200 saccades were sorted from the shortest to the longest length value and then paired, ultimately creating two 1200 survival percent bins. For each of the 1200 survival percent bins, the difference between the high- and low-frequency condition was computed. Finally, the average length of the pair of saccades corresponding to the first survival percent bin in a run of 100 consecutive bins with the positive values of the difference was defined as the divergence point value. The median value across the successful iterations (with a divergence point value) was then defined as the divergence point estimate for that individual. Participants for whom a divergence point value was obtained in more than 50% of iterations were included in the computation of group divergence point estimates.

For the incoming-saccades, as shown in Figures 2C,E, the high- and low-frequency survival curves diverged in a short saccade length, regardless of the DPA procedures being used (the average number of saccades per subject is 16.56 and 20.06 in the high- and low-frequency condition, respectively). Specifically, the Confidence Interval DPA procedure yielded a divergence point estimate (*M* = 1.64 characters, with 95% confidence interval from 1.32 to 1.93 characters). A total of 12.74% of saccades in the high-frequency condition were shorter than the divergence point compared to 14.19% of saccades in the low-frequency condition. Similarly, the Individual Participant DPA procedure produced an average divergence point estimate across individual participants (*M* = 1.70 characters, *SD* = 0.44). In this procedure, 13.32% of saccades in the high-frequency condition and 15.88% of saccades in the low-frequency condition were shorter than the divergence point.

For the outgoing-saccades, as shown in Figures 3C,E, the high- and low-frequency survival curves also diverged in a short saccade length, regardless of the DPA procedures being used (the average number of saccades per subject is 24.22 and 24.58 in the high- and low-frequency condition, respectively). The Confidence Interval DPA procedure yielded a divergence point estimate (*M* = 1.25 characters, with 95% confidence interval from 1.07 to 1.39 characters). A total of 2.86% of saccades in the high-frequency condition were shorter than the divergence point compared to 4.52% of saccades in the low-frequency condition. Similarly, the Individual Participant DPA procedure produced an average divergence point estimate across individual participants (*M* = 1.72 characters, *SD* = 0.69). Altogether, 12.80% of saccades in the high-frequency condition were shorter than the divergence point compared to 18.20% of saccades in the low-frequency condition.

## Computer simulations

In the empirical results, we found that the divergence points for the incoming and outgoing saccades appeared early (or in a short saccade length) and the proportion of saccades longer than the divergence points are large (>>50%), regardless of which DPA procedure we adopted. These empirical results collectively support that the word frequency can appear early in a short saccade length, as the *Gradual-adjusting Account* suggested, rather than later in a long saccade length by the skipping way as the *Slight-skipping Account* suggested. To further examine our results, we run simulations using the Dynamic-adjustment Model of saccade targeting in Chinese reading (Liu et al., 2016). The Dynamic-adjustment Model hypothesized that readers dynamically determine the target position when they decide where to move their eyes in Chinese reading, with the length of a saccade being adjusted to maximize the efficiency of foveal and parafoveal processing. The model aims to provide a qualitative interpretation of saccade target selection rather than a complete word processing and eye-movement control model during Chinese reading. To instantiate and simplify the model, Liu et al. assumed saccade length was a linear function of the parafoveal preview, which was a random deviate sampled from a gamma distribution having a shape parameter, α, and a scale parameter, β (see Equation 1).

(1) $$\begin{array}{l} \text{preview}=\text{Gamma }\left(\alpha ,\beta \right) \end{array}$$

The precise amount of preview could also be modulated by foveal and/or parafoveal processing difficulties (e.g., word's frequency), as specified by Equation (2), where the free parameters η1 and η0 could scale α. It should be noted that to simplify the simulation of the model, we run the simulations for incoming and outgoing saccades separately. Specifically, the frequency of target word was inputted to Equation (2) to represent the parafoveal- and foveal-processing difficulties when simulating incoming and outgoing saccades, respectively. To further simplify the simulation, the weak effect of saccade launch-site distance on preview was also ignored.

(2) $$\begin{array}{l} \alpha =\eta 1\text{frequency}+\eta 0 \end{array}$$

Finally, as assumed by Liu et al., saccade length was modulated by preview through a free parameter λ (as specified by Equation (3). The best fitting parameter values and the procedure used to search them are described in the Appendix).

(3) $$\begin{array}{r} \text{length}=\lambda \text{ preview}\\ =\lambda \text{Gamma }\left(\alpha ,\beta \right)\\ =\text{Gamma }\left(\eta 1\text{frequency}+\eta 0,\lambda \beta \right) \end{array}$$

The simulation results are displayed in the right panels of Figures 2, 3 to facilitate comparison with empirical results. As seen from both figures, the survival analysis results from the simulations showed a qualitatively similar pattern with the empirical survival analysis results. More importantly, similar to the divergence points found in the empirical datasets, the divergence points found in the simulated dataset also appeared early (or in a short saccade length), and the proportion of saccades longer than the divergence points were still large. Specifically, for the incoming saccades (the average number of saccades per subject is 26.61 and 26.11 in the high- and low-frequency condition, respectively), the Confidence Interval DPA procedure yielded a divergence point estimate (*M* = 1.36 characters, with 95% confidence interval from 1.07 to 1.50 characters). A total of 1.89% of saccades in the high-frequency condition were shorter than the divergence point compared to 3.67% of saccades in the low-frequency condition. Similarly, the Individual Participant DPA procedure produced an average divergence point estimate across individual participants (*M* = 1.60 characters, *SD* = 0.41). A total of 7.14% of saccades in the high-frequency condition were shorter than the divergence point compared to 12.12% of saccades in the low-frequency condition.

Finally, for the outgoing saccades (the average number of saccades per subject is 38.11 and 36.47 in the high- and low-frequency condition, respectively), the Confidence Interval DPA procedure yielded a divergence point estimate (*M* = 1.21 characters, with 95% confidence interval from 1.11 to 1.29 characters). A total of 0.43% of saccades in the high-frequency condition were shorter than the divergence point compared to 2.44% of saccades in the low-frequency condition. Again, the individual participant DPA procedure produced an average divergence point estimate across individual participants (*M* = 1.56 characters, *SD* = 0.42). A total of 9.32% of saccades in the high-frequency condition were shorter than the divergence point compared to 4.65% of saccades in the low-frequency condition.

## General discussion

In this paper, we used survival-curve analysis to examine how the frequency of target words influences the saccades entering and exiting it. To check the reliability of the results, we used the Confidence Interval DPA and Individual Participant DPA procedures to analyze the data from one empirical experiment and simulations based on the Dynamic-adjustment Model. We found word frequency effect diverged early in the distribution of saccade lengths, regardless of the DPA procedures or data being used. For incoming saccades, the word-frequency effect first appeared at 1.36–1.70 character spaces from the launching of saccades, with 86.68–98.11% of saccades entering high-frequency words and 84.12–96.23% of saccades entering low-frequency words being larger than the divergence points, respectively. For outgoing saccades, the word-frequency effect first appeared at 1.21–1.72 character spaces from the launching of saccades, with 87.20–99.57% of saccades leaving high-frequency words and 82.80–97.56% of saccades leaving low-frequency words being larger than the divergence points, respectively. As an extension of previous work (e.g., Liu et al., 2016), the current analyses provide additional evidence to support that the frequency of target words can influence saccades into and out of these target words gradually (i.e., the *Gradual-adjusting Account*) rather than influencing a few long saccades to skip the next word (i.e., the *Slight-skipping Account*) in Chinese reading. Further, the consistent results between the empirical and simulated data also support the Dynamic-adjustment Model.

It should be noted that the survival analysis results are also consistent with the documented results that the lexical and sub-lexical properties of upcoming words can affect saccade targeting during the reading of alphabetic language. For example, saccades tend to be located at the beginning of the upcoming word if they contain orthographic irregularities or unfamiliar spelling patterns (Radach et al., 2004; Plummer and Rayner, 2012). Similarly, the initial fixation locations on Finnish compound words are influenced by the frequency of the first morphemic constituent of the word (Hyönä and Pollatsek, 1998, 2000). These findings collectively indicate that lexical and sub-lexical information in the fovea and parafovea affect saccade lengths.

More importantly, the survival analysis results from the empirical data were consistent with the simulated data, providing more evidence to support the Dynamic-adjustment model, which assumed that the amount of parafoveal processing can modulate saccade length in Chinese reading (Yan et al., 2010; Liu et al., 2016, in press). As presented in this article, Liu et al. (2016) has instantiated this theory in a computational model by assuming that saccade length is linearly related to the extent of preview. It is interesting that this simple model can explain saccade behaviors with the highest quantitative fitness and fewest parameters. For instance, the model can fit the relationship between the pre-target launch site and the landing site related to target words, the probabilities of refixating pre-target words, the fixation on or skipping of target words, and the word frequency effect on the incoming saccade length as well as the corresponding landing positions (for detailed information, see Figures 2–5 in Liu et al., 2016). In a new endeavor, Liu et al. (in press) have incorporated the launch-site and launch-word frequency effect into this novel model. This model can also fit the aforementioned saccade patterns (i.e., the relationship between launch site and landing site, the probabilities of fixating various words) with the highest quantitative accuracy. In addition to these previous results, our findings that early divergence points and a large proportion of saccades are longer than the divergence points provide new evidence to support the Dynamic-adjustment Model. To the best of our knowledge, the Dynamic-adjustment Model is the first formal model that can fit these empirical patterns with the highest quantitative accuracy and easiest theoretical assumption (e.g., fewest parameters) during Chinese reading.

Lastly, we used the survival analysis technique to provide more precise evidence to support the word frequency effect on forward saccades gradually, further supporting the Dynamic-adjustment Model. Combined with prior results (e.g., Liu et al., 2016), these findings have established the “benchmarks” for any future computational models of eye-movement control during the reading of Chinese. To understand these complex eye movement controls, it is obvious that more empirical and modeling work should be conducted to examine how local processing in the fovea and parafovea dynamically influence eye movements.

## Ethics statement

The empirical data from a published paper (Liu et al., 2016).

## Author contributions

Y. Liu and RH designed research; Y. Liu, RH and Y. Li performed research; Y. Liu, analyzed data; and Y. Liu, RH, and DG, wrote the paper.

## Funding

This research was supported by grants from the National Natural Science Foundation of China (31500890 & 31371028) to the first and last authors.

### Conflict of interest statement

The authors declare that the research was conducted in the absence of any commercial or financial relationships that could be construed as a potential conflict of interest.
