# Supplementary material for: The Word Frequency Effect on Saccade Targeting during Chinese Reading: Evidence from a Survival Analysis of Saccade Length
Source: Front Psychol. 2017 Feb 6;8:116. doi: 10.3389/fpsyg.2017.00116 (PMC5292409; doi:10.3389/fpsyg.2017.00116)
Supplement: Supplementary file 1 [file DataSheet1.docx]

**Appendix**

The expected value of Equation 3 is λβ(η1*frequency* + η0), which equals the value predicted using the mean progressive saccade length from each saccade launch region. Therefore, two groups of parameters, λβη1 and λβη0, the coefficients for a regression equation for the progressive saccade length, are calculated by using target-word frequency as a predictor variable (i.e., low-frequency = 1, high-frequency = 2). Additionally, because the variance (i.e., associated with saccadic error) of equation 3 is given by the quantity λ^2^β^2^(η1*frequency* + η0), the parameter pair λβ can be estimated by using the empirical distribution of fixations on the target words or on the words immediately following the target words for incoming and outgoing saccades, respectively. The procedure is performed separately for the incoming and outgoing saccades as well as for the low- and high-frequency conditions. The best-fitting parameters used for incoming and outgoing saccades by high- and low-frequency conditions are listed in Tables A1 and A2, respectively. Finally, we simulated the 36 subjects by using these best-fitting parameters for incoming and outgoing saccades, respectively. For each subject, we ran 80 trials for the high-frequency and low-frequency conditions, respectively. These data were then used for the survival analysis.

**Table A1**. The best-fitting parameters used for simulating incoming saccades.

| Target-frequency | η0 | η1 | αβ |
| --- | --- | --- | --- |
| Low | 11.47 | 0.55 | 0.20 |
| High | 9.63 | 0.46 | 0.24 |

**Table A2.** The best-fitting parameters used for simulating outgoing saccades.

| Target-frequency | η0 | η1 | αβ |
| --- | --- | --- | --- |
| Low | 7.51 | 0.72 | 0.29 |
| High | 9.53 | 0.91 | 0.23 |
